# Double agent indole-3-acetic acid: mechanistic analysis of indole-3-acetaldehyde dehydrogenase AldA that synthesizes IAA, an auxin that aids bacterial virulence

**DOI:** 10.1042/BSR20210598

**Published:** 2021-08-24

**Authors:** Ateek Shah, Yamini Mathur, Amrita B. Hazra

**Affiliations:** 1 Department of Chemistry, Indian Institute of Science Education and Research, Dr. Homi Bhabha Road, Pashan, Pune 411008, India; 2 Department of Biology, Indian Institute of Science Education and Research, Dr. Homi Bhabha Road, Pashan, Pune 411008, India

**Keywords:** aldehyde dehydrogenase, auxins, cofactor isomerization, host-pathogen interactions, indole-3-acetic acid (IAA), Pseudomonas

## Abstract

The large diversity of organisms inhabiting various environmental niches on our planet are engaged in a lively exchange of biomolecules, including nutrients, hormones, and vitamins. In a quest to survive, organisms that we define as pathogens employ innovative methods to extract valuable resources from their host leading to an infection. One such instance is where plant-associated bacterial pathogens synthesize and deploy hormones or their molecular mimics to manipulate the physiology of the host plant. This commentary describes one such specific example—the mechanism of the enzyme AldA, an aldehyde dehydrogenase (ALDH) from the bacterial plant pathogen *Pseudomonas syringae* which produces the plant auxin hormone indole-3-acetic acid (IAA) by oxidizing the substrate indole-3-acetaldehyde (IAAld) using the cofactor nicotinamide adenine dinucleotide (NAD^+^) (*Bioscience Reports* (2020) **40**(12), https://doi.org/10.1042/BSR20202959). Using mutagenesis, enzyme kinetics, and structural analysis, Zhang et al. established that the progress of the reaction hinges on the formation of two distinct conformations of NAD(H) during the reaction course. Additionally, a key mutation in the AldA active site ‘aromatic box’ changes the enzyme’s preference for an aromatic substrate to an aliphatic one. Our commentary concludes that such molecular level investigations help to establish the nature of the dynamics of NAD(H) in ALDH-catalyzed reactions, and further show that the key active site residues control substrate specificity. We also contemplate that insights from the present study can be used to engineer novel ALDH enzymes for environmental, health, and industrial applications.

The immense diversity of life on earth is supported by an intricate give-and-take between organisms that inhabit various environmental niches. Most known ecosystems host multiple species that engage in an enduring exchange of biomolecules which are classified as positive or negative interactions (beneficial/neutral or detrimental to one or more of the species, respectively), and are precisely tailored to meet the nutritional and proliferative needs of the organisms [1,2]. Some of the most fascinating examples of interactions among species are where one organism is host to another which is a pathogen, that is, the pathogenic species acquires nutrients and proliferates, while the host suffers the consequences of the infection. For example, the relationship between *Phaeobacter gallaeciensis*, a marine bacterium and *Emiliania huxleyi*, a marine microalga is proposed to be a mutual exchange with the algae providing dimethylsulfoniopropionate (a carbon and sulfur source) to the bacteria and the bacteria providing growth hormones (phenylacetic acid) and antibiotics (tropodithietic acid) in return. In an intriguing turn of events, once *E. huxleyi* begins to senesce, it releases *p*-coumaric acid which is sensed by *P. gallaeciensis. P. gallaeciensis* then synthesizes roseobacticides to kill the algae and scavenge nutrients from its victim for its growth [3]. In the natural world, there are several innovative means that pathogens use to gain entry into the host. Rabies virus, a member of the Rhabdoviridae family, enters into nerve ending-rich muscle and tissues through the bite of an animal and then proliferates and travels via nerve cells to the brain causing hydrophobia, hallucinations, and eventually death [4]. The blast disease of rice is caused by the fungus *Magnaporthe oryzae* which produces a flattened, hyphal cell from the germinating spore with an infection peg that penetrates the host causing brown diamond-shape lesions on the rice leaves [5]. Interestingly, many plant-associated microbes synthesize a plant hormone or its close mimic to gain entry and establish themselves by modulating the host plant’s metabolism as well as their own virulence genes [6–8]. An extensively studied example of hormone-mediated plant–microbe pathogenesis is the crown gall disease caused by the Gram-negative soil bacterium, *Agrobacterium tumefaciens.* The bacterium senses chemoattractants such as phenolic compounds released from wounded regions on the plants and initiates transfer of bacterial DNA to the host chromosome which hijacks the metabolism in the infected plant cells [9]. *A. tumefaciens* harbors a specialized tumor-inducing (Ti) plasmid which encodes an array of genes that aid in its transfer, replication, and in overall plant–microbe pathogenesis [10]. The T-DNA region of the Ti plasmid is transferred to the plant nuclear DNA, which initiates the biosynthesis of plant hormones auxin and cytokinins via the *iaaM* and *iaaH*, and *ipt* pathways, respectively, which are absent from the host plant (Figure 1). This results in uncontrolled cell proliferation and subsequently crown gall tumors [10].

**Figure 1:**
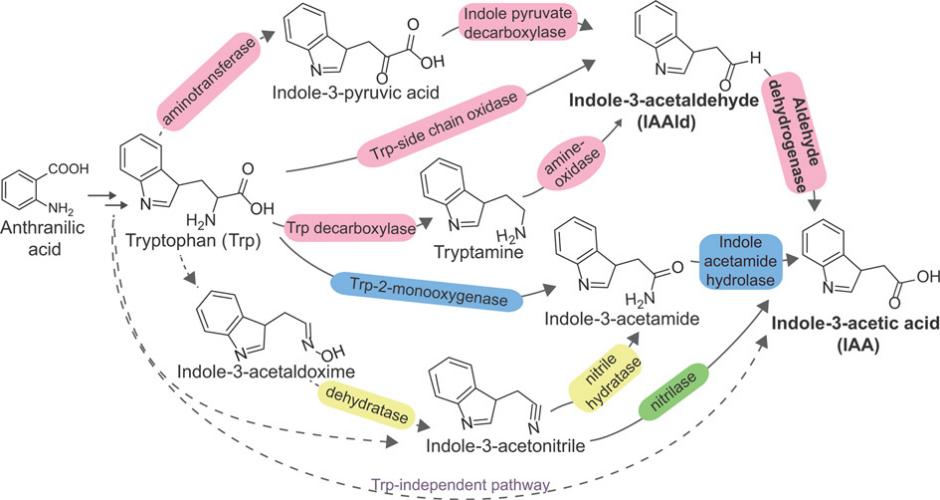
Bacterial biosynthesis of auxin Certain plant-associated bacteria can produce auxin indole-3-acetic acid (IAA) from tryptophan (Trp) as a precursor using one or more than one of the routes shown here. Among the known Trp-dependent pathways, three routes involving (i) aminotransferase and indole-3-pyruvate decarboxylase, (ii) Trp-side chain oxidase, and (iii) Trp deacarboxylase and an amine-oxidase converge at indole-3-acetaldehyde (IAAld) which is then oxidized by aldehyde dehydrogenase (ALDH) to yield IAA *(enzymes shown in pink*). Another prominent route involves the intermediate indole-3-acetamide which can be synthesized directly from tryptophan by tryptophan 2-monooxygenase (*iaaM*), and then converted into IAA via the enzyme indole acetamide hydrolase (*iaaH*) *(enzymes shown in blue)*. Alternately, indole-3-acetamide is synthesized via a two-step reaction involving a predicted dehydratase and nitrile hydratase *(enzymes shown in yellow)* followed by conversion into IAA as mentioned previously. Finally, some bacteria appear to use the lesser studied Trp-independent routes, though the precursors appear to be derived from Trp biosynthesis intermediates [16,17]. For example, indole-3-acetonitrile, derived from a precursor of Trp yield IAA in a single-step reaction catalyzed by the nitrilase enzyme *(enzyme shown in green)*. The reactions for which genetic and biochemical evidence of the enzymes involved are yet to discovered are shown with dashed arrows. *Pseudomonas syringae* DC3000 genome possesses genes for amine oxidase, nitrilase, indole acetamide hydrolase, ALDH, and a putative monooxygenase [19]. The enzymes shown with dashed arrows have some biochemical evidence however, the encoding genes are yet unknown. Figure adapted from Spaepen and Vanderleyden (2011) [15] and Duca et al. (2014) [18].

Another intriguing example is that of *Pseudomonas syringae*, a bacterial plant pathogen that manipulates hormone signaling as a means of breaking the host plant defense [8,11]. It gains entry into the tomato plant by producing the phytotoxin coronatine, a molecular mimic of the plant hormone jasmonic acid-isoleucine, resulting in the bacterial speck disease [7,12,13]. *P. syringae* also synthesizes the auxin indole-3-acetic acid (IAA), a common growth hormone synthesized by plants for cell enlargement, division, and differentiation and uses it to establish itself in its plant host [11,14]. IAA can be synthesized in bacteria from tryptophan as a precursor via five distinct routes, and also via tryptophan-independent pathways which mostly yet remain to be characterized in detail [15–18] (Figure 1).

Three of the tryptophan-dependent pathways result in the formation of a common intermediate indole-3-acetaldehyde (IAAld) which is oxidized by an aldehyde dehydrogenase (ALDH) to IAA. ALDHs are housekeeping enzymes that play a major role in the detoxification of reactive aldehydes by converting them into their corresponding carboxylic acid [20–23]. ALDHs are known to participate in important functions such as polyamine catabolism [24], ethanol metabolism [25,26], xenobiotic metabolism [27–29], and plant cell wall ester synthesis [30,31], yet, many ALDH-catalyzed reactions remain to be characterized in detail in bacteria.

In 2018, the Kunkel laboratory reported the identification of six putative ALDHs in the pathogenic *P*. *syringae* pv. tomato strain DC3000 [11]. Biochemical analysis of these six homologs revealed that AldA, AldB, and AldC were capable of producing IAA from IAAld. Further, a high-resolution crystal structure of wildtype AldA was obtained. Additionally, 42 classes of genes encoding putative ALDHs in various *Pseudomonas* species have been recently reported, expanding the scope of this family of enzymes in bacterial metabolism [23]. The role of AldA was established to be a nicotinamide adenine dinucleotide (NAD^+^)-dependent IAAld dehydrogenase that produces IAA [11]. In 2020, biochemical and structural characterization of AldC by Lee et al. revealed it to be a long-chain aliphatic ALDH [32]. IAA produced by *P. syringae* has been reported to promote the virulence in *Arabidopsis thaliana* by two different mechanisms—one, it up-regulates the virulence gene expression in the bacterium, and second, it suppresses salicylic acid-mediated plant defenses by activating auxin signaling in the host plant (via the TIR1/AFB auxin co-receptor system) [8,11]. Studies with *tir1/afb* mutants show accumulation of IAA in *A. thaliana*, resulting in the deregulation of plant host signaling. This results in increased host susceptibility to the pathogen’s entry [8] (Figure 2). Targeting the arsenal of enzymes that *P. syringae* uses for infection is one the strategies to suppress its pathogenicity. To this end, Zhang et al. have recently reported a detailed study of the enzyme AldA to provide a glimpse into its molecular mechanism of action [33].

**Figure 2:**
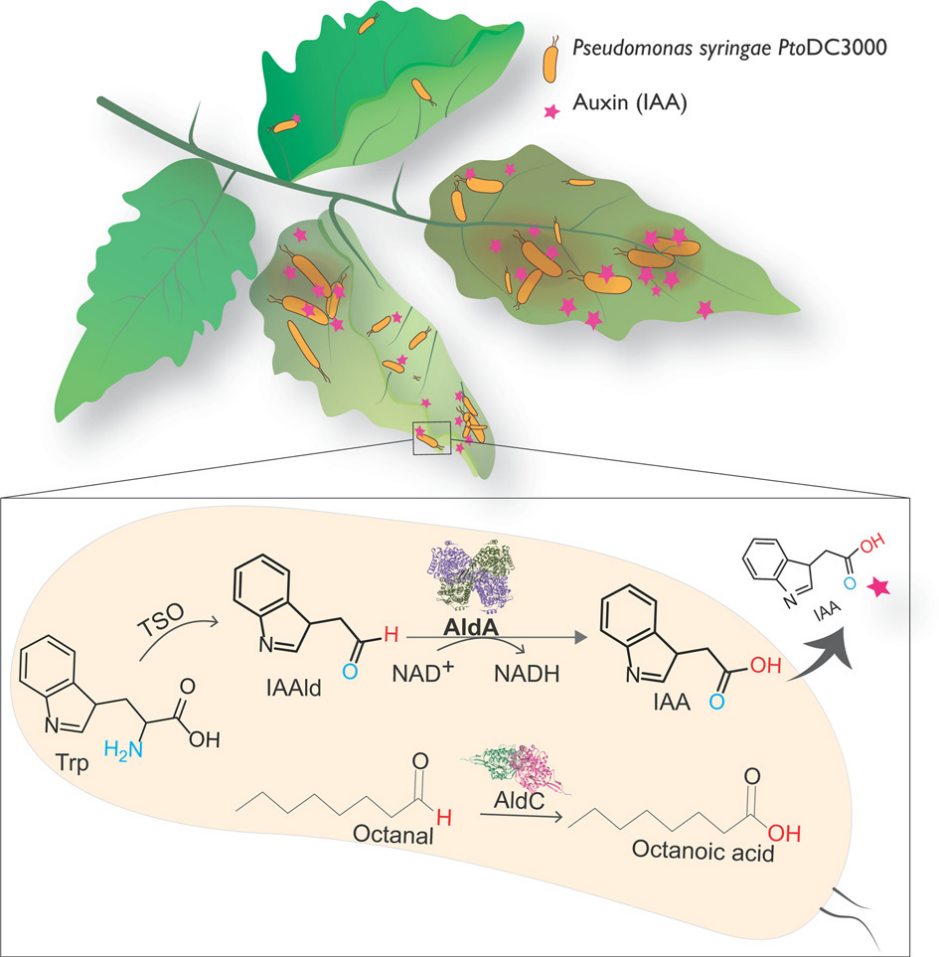
Plant pathogen *P. syringae pv. tomato strain* DC3000 synthesizes the auxin IAA, a plant growth hormone that facilitates its entry into the growing plant, and causing wilting and discoloration of the infected parts AldA is an IAAld dehydrogenase in *P. syringae* DC3000 which catalyzes the oxidation of IAAld to IAA coupled with reduction of NAD^+^ cofactor. Please refer to Figure 1 from Zhang et al. (2020) [33] for the detailed mechanism. AldC, another ALDH found in *P. syringae* oxidizes a range of long-chain aliphatic aldehydes such as octanal to the corresponding acids. Such aliphatic molecules have been hypothesized to assist microbes in gaining entry into the host cell and also to act as nutrients for their proliferation [32,34].

The reaction mechanism of AldA can be broadly described in two parts. First, an active site catalytic residue Cys^302^ forms a thiohemiacetal intermediate with the IAAld which is followed by a hydride transfer from the aldehyde to the NAD^+^ cofactor resulting in a thioacyl-enzyme intermediate. Second, the active site residue Glu^267^ activates a water molecule for the hydrolysis of the intermediate resulting in the release of the carboxylic acid product and the reduced cofactor NADH [11]. Zhang et al. created mutants of these two catalytic residues and other substrate-binding site residues to study the mechanism in detail using kinetic and structural tools [33].

The activity of the wildtype and mutants of AldA were tested with IAAld which is the physiological substrate. As expected, enzyme assays using the AldA catalytic residue Cys^302^ and Glu^267^ mutants (Cys^302^Ala, Glu^267^Gln, Glu^267^Ala) showed no detectable activity. Noticeably, mutations in the aliphatic and aromatic residues lining the substrate-binding site of AldA were especially detrimental to the binding of IAA and lowered the catalytic efficiency significantly. These mutants were also tested for activity with octanal, the preferred substrate of AldC. Interestingly, the substitution of Phe^169^ with Trp (the corresponding residue found in AldC at that position) resulted in a higher turnover with octanal as compared with IAAld, thus leading to a reversal in the substrate preference and catalytic efficiency of the AldA Phe^169^Trp mutant [33]. Such a shift in the preference for chemically distinct substrates arising from a single point mutation indicates that a small number of precise changes in the active site are likely responsible for the extensive biochemical scope of enzymes in the ALDH enzyme superfamily.

An interesting feature observed in the mechanism of ALDH enzymes is cofactor isomerization wherein after the hydride transfer, the nicotinamide ring of the NAD(H) cofactor tilts away from the catalytic cysteine and glutamate residues [35,36]. Zhang et al., captured the two orientations of the cofactor NAD^+^ in their crystal structure analysis of AldA [33]. The ‘extended’ conformation where the nicotinamide half of the NAD^+^ cofactor is buried in the active site, with the nicotinamide in close proximity to Cys^302^ and Glu^267^, and the ribose part forming hydrogen bonds with another active site residue Glu^401^ was found in the wildtype AldA enzyme. In contrast, a ‘contracted’ conformation where the NAD^+^ nicotinamide ring is pulled away from the catalytic site and the nicotinamide-ribose orients towards an active site residue Gln^349^ was found in the catalytically dead AldA Cys^302^Ala mutant. This movement allows each half-reaction to occur efficiently, the hydride transfer part followed by the final hydrolysis of the enzyme–product complex which marks the completion of the reaction. A comparison of these two structures provides a glimpse into the dynamic interactions of the AldA active site residues with NAD(H) through the course of reaction, and highlights the molecular significance of these interactions in the mechanism of ALDH.

This series of investigations from the Kunkel and Jez labs on *P. syringae* ALDHs have opened several avenues in the study of bacterial IAA biosynthesis and their role in plant–microbe interactions. For example, the three characterized ALDH homologs AldA, AldB, and AldC were found via bioinformatics-based searches and share sufficient sequence identity [11]. However, their biochemical characterization show that they have different substrate preferences which would lead to them playing distinct functional roles in the cells. Obtaining a molecular level picture of the differences between the ALDH homologs via detailed biochemical characterization with the enzymes and their mutants would contribute to a bigger picture understanding of their function and physiological roles in *P. syringae* pathogenesis. Further, in addition to ALDHs that synthesize IAA from IAAld, the *P. synringae* DC3000 genome also possesses genes to convert tryptamine, indole-3-acetamide, and indole-3-acetonitrile into IAA [19] (Figure 1). Additionally, a homolog of lysine monooxygenase has been hypothesized to synthesize indole-3-acetamide from tryptophan [11]. Functional characterization of these gene products are required to understand the flux of tryptophan to produce IAA and validate all the operating pathways for auxin biosynthesis in the bacterium. Given the agro-biotechnological and ecological importance of *P. syringae* [37,38], mechanistic enzymology studies such as these are important steps for in-depth understanding of host–microbe interaction and to keep its pathogenicity in check.

## Conclusion

IAA plays a vital role in plant growth, and doubles up as an agent that regulates bacterial virulence, thus aiding the entry of *P. syringae* and other microbial plant pathogens into plants. Specifically inhibiting IAA biosynthesis in pathogenic bacteria may therefore act as an effective strategy to reduce widespread microbial infections in plants. Through the characterization and detailed mechanistic study of *P. syringae* AldA, the Jez and Kunkel laboratories took a step in this direction [11,33]. The unique NAD(H) cofactor isomerization that occurs in ALDH enzymes (and also seen in the *P. syringae* AldA structures in this work) is especially attractive—analyzing this step not only broadens our basic understanding of how NAD(H) functions, but also provides a prospective target for inhibiting the reaction at the halfway point and inactivating the enzyme. Finally, the ALDH family appears to accommodate a variety of aldehyde substrates [21–23,25,39–41]. Analysis of the active site ‘aromatic box’ in AldA allowed for a single amino acid substitution that altered the substrate preference from an indole-based substrate (aromatic) to an octanal (aliphatic) with higher catalytic efficiency [33]. This opens up the possibility of directed evolution and enzyme engineering of the ALDH superfamily members to design enzymes that can aid in the detoxification of a wide range of aldehyde substrates in environmental, health, and industrial applications.

## Abbreviations

ALDH: aldehyde dehydrogenase
IAA: indole-3-acetic acid
IAAld: indole-3-acetaldehyde
NAD^+^: nicotinamide adenine dinucleotide
T-DNA: transfer-DNA
Ti: tumor-inducing

## References

[1] Tshikantwa T.S., Ullah M.W., He F. and Yang G. (2018) Current trends and potential applications of microbial interactions for human welfare. Front. Microbiol. 9, 1156 10.3389/fmicb.2018.01156 29910788 PMC5992746

[2] Braga R.M., Dourado M.N. and Araújo W.L. (2016) Microbial interactions: ecology in a molecular perspective. Brazilian J. Microbiol. 47, 86–98 10.1016/j.bjm.2016.10.005 27825606 PMC5156507

[3] Seyedsayamdost M.R., Case R.J., Kolter R. and Clardy J. (2011) The Jekyll-and-Hyde chemistry of *Phaeobacter gallaeciensis*. Nat. Chem. 3, 331–335 10.1038/nchem.1002 21430694 PMC3376411

[4] Yousaf M.Z., Qasim M., Zia S., Khan M.R., Ashfaq U.A. and Khan S. (2012) Rabies molecular virology, diagnosis, prevention and treatment. Virol. J. 9, 50 10.1186/1743-422X-9-50 22348291 PMC3307483

[5] Shahriar S.A., Imtiaz A.A., Hossain M.B., Husna A. and Eaty M.N.K. (2020) Review: Rice blast disease. Annu. Res. Rev. Biol. 35, 50–64 10.9734/arrb/2020/v35i130180

[6] Jones J.D.G. and Dangl J.L. (2006) The plant immune system. Nature 444, 323–329 10.1038/nature05286 17108957

[7] Xin X.F. and He S.Y. (2013) *Pseudomonas syringae* pv. tomato DC3000: a model pathogen for probing disease susceptibility and hormone signaling in plants. Annu. Rev. Phytopathol. 51, 473–498 10.1146/annurev-phyto-082712-102321 23725467

[8] Djami-Tchatchou A.T., Harrison G.A., Harper C.P., Wang R., Prigge M.J., Estelle M. et al. (2020) Dual role of auxin in regulating plant defense and bacterial virulence gene expression during *Pseudomonas syringae* PtoDC3000 pathogenesis. Mol. Plant Microbe Interact. 33, 1059–1071 10.1094/MPMI-02-20-0047-R 32407150 PMC7810136

[9] Escobar M.A. and Dandekar A.M. (2003) *Agrobacterium tumefaciens* as an agent of disease. Trends Plant Sci. 8, 380–386 10.1016/S1360-1385(03)00162-6 12927971

[10] Gordon J.E. and Christie P.J. (2014) The Agrobacterium Ti plasmids. Microbiol. Spect. 2, 1–18, PLAS-0010–2013 10.1128/microbiolspec.PLAS-0010-2013 PMC4292801 25593788

[11] McClerklin S.A., Lee S.G., Harper C.P., Nwumeh R., Jez J.M. and Kunkel B.N. (2018) Indole-3-acetaldehyde dehydrogenase-dependent auxin synthesis contributes to virulence of *Pseudomonas syringae* strain DC3000. PLoS Pathog. 14, e1006811 10.1371/journal.ppat.1006811 29293681 PMC5766252

[12] Preston G.M. (2000) *Pseudomonas syringae* pv. tomato: the right pathogen, of the right plant, at the right time. Mol. Plant Pathol. 1, 263–275 10.1046/j.1364-3703.2000.00036.x 20572973

[13] Katsir L., Schilmiller A.L., Staswick P.E., He S.Y. and Howe G.A. (2008) COI1 is a critical component of a receptor for jasmonate and the bacterial virulence factor coronatine. Proc. Natl. Acad. Sci. U.S.A. 105, 7100–7105 10.1073/pnas.0802332105 18458331 PMC2383947

[14] Casanova-Sáez R., Mateo-Bonmatí E. and Ljung K. (2021) Auxin metabolism in plants. Cold Spring Harb. Perspect. Biol. 13, a039867 10.1101/cshperspect.a039867 33431579 PMC7919392

[15] Spaepen S. and Vanderleyden J. (2011) Auxin and plant-microbe interactions. Cold Spring Harb. Perspect. Biol. 3, a001438 10.1101/cshperspect.a001438 21084388 PMC3062209

[16] Prinsen E. (1993) *Azospirillum brasilense* indole-3-acetic acid biosynthesis: evidence for a non-tryptophan dependent pathway. Mol. Plant Microbe Interact. 6, 609 10.1094/MPMI-6-609

[17] Ahmad E., Sharma S.K. and Sharma P.K. (2021) Deciphering operation of tryptophan-independent pathway in high indole-3-acetic acid (IAA) producing *Micrococcus aloeverae* DCB-20. FEMS Microbiol. Lett. 367, fnaa190 10.1093/femsle/fnaa190 33201985

[18] Duca D., Lorv J., Patten C.L., Rose D. and Glick B.R. (2014) Indole-3-acetic acid in plant-microbe interactions. Antonie Van Leeuwenhoek 106, 85–125 10.1007/s10482-013-0095-y 24445491

[19] Kanehisa M., Sato Y., Kawashima M., Furumichi M. and Tanabe M. (2016) KEGG as a reference resource for gene and protein annotation. Nucleic Acids Res. 44, 457–462 10.1093/nar/gkv1070 PMC4702792 26476454

[20] Yoshida A., Rzhetsky A., Hsu L.C. and Chang C. (1998) Human aldehyde dehydrogenase gene family. Eur. J. Biochem. 251, 549–557 10.1046/j.1432-1327.1998.2510549.x 9490025

[21] Sophos N.A. and Vasiliou V. (2003) Aldehyde dehydrogenase gene superfamily: the 2002 update. Chem. Biol. Interact. 143–144, 5–22 10.1016/S0009-2797(02)00163-1 12604184

[22] Brocker C., Vasiliou M., Carpenter S., Carpenter C., Zhang Y., Wang X. et al. (2013) Aldehyde dehydrogenase (ALDH) superfamily in plants: gene nomenclature and comparative genomics. Planta 237, 189–210 10.1007/s00425-012-1749-0 23007552 PMC3536936

[23] Riveros-Rosas H., Julián-Sánchez A., Moreno-Hagelsieb G. and Muñoz-Clares R.A. (2019) Aldehyde dehydrogenase diversity in bacteria of the *Pseudomonas* genus. Chem. Biol. Interact. 304, 83–87 10.1016/j.cbi.2019.03.006 30862475

[24] Brocker C., Lassen N., Estey T., Pappa A., Cantore M., Orlova V.V. et al. (2010) Aldehyde dehydrogenase 7A1 (ALDH7A1) is a novel enzyme involved in cellular defense against hyperosmotic stress. J. Biol. Chem. 285, 18452–18463 10.1074/jbc.M109.077925 20207735 PMC2881771

[25] Klyosov A.A. (1996) Kinetics and specificity of human liver aldehyde dehydrogenases toward aliphatic, aromatic, and fused polycyclic aldehydes†. Biochemistry 35, 4457–4467 10.1021/bi9521102 8605195

[26] Steinmetz C.G., Xie P., Weiner H. and Hurley T.D. (1997) Structure of mitochondrial aldehyde dehydrogenase: the genetic component of ethanol aversion. Structure 5, 701–711 10.1016/S0969-2126(97)00224-4 9195888

[27] Coitinho J.B., Pereira M.S., Costa D.M.A., Guimarães S.L., Araújo S.S., Hengge A.C. et al. (2016) Structural and kinetic properties of the aldehyde dehydrogenase nahf, a broad substrate specificity enzyme for aldehyde oxidation. Biochemistry 55, 5453–5463 10.1021/acs.biochem.6b00614 27580341

[28] Crabo A.G., Singh B., Nguyen T., Emami S., Gassner G.T. and Sazinsky M.H. (2017) Structure and biochemistry of phenylacetaldehyde dehydrogenase from the *Pseudomonas putida* S12 styrene catabolic pathway. Arch. Biochem. Biophys. 616, 47–58 10.1016/j.abb.2017.01.011 28153386 PMC5318141

[29] Zahniser M.P.D., Prasad S., Kneen M.M., Kreinbring C.A., Petsko G.A., Ringe D. et al. (2017) Structure and mechanism of benzaldehyde dehydrogenase from *Pseudomonas putida* ATCC 12633, a member of the Class 3 aldehyde dehydrogenase superfamily. Protein Eng. Des. Select. 30, 273–280 10.1093/protein/gzx015 PMC5421609 28338942

[30] Nair R.B., Bastress K.L., Ruegger M.O., Denault J.W. and Chapple C. (2004) The *Arabidopsis thaliana* REDUCED EPIDERMAL FLUORESCENCE1 gene encodes an aldehyde dehydrogenase involved in ferulic acid and sinapic acid biosynthesis. Plant Cell 16, 544–554 10.1105/tpc.017509 14729911 PMC341923

[31] Bosch M., Mayer C.D., Cookson A. and Donnison I.S. (2011) Identification of genes involved in cell wall biogenesis in grasses by differential gene expression profiling of elongating and non-elongating maize internodes. J. Exp. Bot. 62, 3545–3561 10.1093/jxb/err045 21402660 PMC3130177

[32] Lee S.G., Harline K., Abar O., Akadri S.O., Bastian A.G., Chen H.Y.S. et al. (2020) The plant pathogen enzyme AldC is a long-chain aliphatic aldehyde dehydrogenase. J. Biol. Chem. 295, 13914–13926 10.1074/jbc.RA120.014747 32796031 PMC7535917

[33] Zhang K., Lee J.S., Liu R., Chan Z.T., Dawson T.J., de Togni E.S. et al. (2020) Investigating the reaction and substrate preference of indole-3-acetaldehyde dehydrogenase from the plant pathogen *Pseudomonas syringae* PtoDC3000. Biosci. Rep. 40, BSR20202959 10.1042/BSR20202959 33325526 PMC7745063

[34] Crouzet J., Arguelles-Arias A., Dhondt-Cordelier S., Cordelier S., Pršić J., Hoff G. et al. (2020) Biosurfactants in plant protection against diseases: Rhamnolipids and Lipopeptides case study. Front. Bioeng. Biotechnol. 8, 1014 10.3389/fbioe.2020.01014 33015005 PMC7505919

[35] Perez-Miller S.J. and Hurley T.D. (2003) Coenzyme isomerization is integral to catalysis in aldehyde dehydrogenase. Biochemistry 42, 7100–7109 10.1021/bi034182w 12795606

[36] Talfournier F., Pailot A., Stinès-Chaumeil C. and Branlant G. (2009) Stabilization and conformational isomerization of the cofactor during the catalysis in hydrolytic ALDHs. Chem. Biol. Interact. 178, 79–83 10.1016/j.cbi.2008.10.045 19028478

[37] Xin X.F., Kvitko B. and He S.Y. (2018) *Pseudomonas syringae:* what it takes to be a pathogen. Nat. Rev. Microbiol. 16, 316–328 10.1038/nrmicro.2018.17 29479077 PMC5972017

[38] Morris C.E., Monteil C.L. and Berge O. (2013) The life history of *Pseudomonas syringae:* linking agriculture to earth system processes. Annu. Rev. Phytopathol. 51, 85–104 10.1146/annurev-phyto-082712-102402 23663005

[39] Wang M.F., Han C.L. and Yin S.J. (2009) Substrate specificity of human and yeast aldehyde dehydrogenases. Chem. Biol. Interact. 178, 36–39 10.1016/j.cbi.2008.10.002 18983993

[40] Pemberton T.A. and Tanner J.J. (2013) Structural basis of substrate selectivity of Δ1-pyrroline-5-carboxylate dehydrogenase (ALDH4A1): semialdehyde chain length. Arch. Biochem. Biophys. 538, 34–40 10.1016/j.abb.2013.07.024 23928095 PMC3915059

[41] Riveros-Rosas H., González-Segura L., Julián-Sánchez A., Díaz-Sánchez Á.G. and Muñoz-Clares R.A. (2013) Structural determinants of substrate specificity in aldehyde dehydrogenases. Chem. Biol. Interact. 202, 51–61 10.1016/j.cbi.2012.11.015 23219887

